# X-ray guided anatomy-based fitting: The validity of OTOPLAN

**DOI:** 10.1371/journal.pone.0313567

**Published:** 2024-11-15

**Authors:** Asma Alahmadi, Yassin Abdelsamad, Ahmed Hafez, Abdulrahman Hagr

**Affiliations:** 1 King Abdullah Ear Specialist Center (KAESC), King Saud Medical City, King Saud University, Riyadh, Saudi Arabia; 2 Research Department, MED-EL GmbH, Riyadh, Saudi Arabia; UFPE: Universidade Federal de Pernambuco, BRAZIL

## Abstract

**Background:**

Anatomy-based fitting (ABF) for cochlear implant users is a new era that seeks improved outcomes. Recently, different imaging modalities, such as plain X-rays, have been proposed to build the ABF as an alternative to the computed tomography (CT) scan. This study aimed to assess the feasibility and validity of OTOPLAN^®^ software in building ABF using plain X-ray imaging.

**Patients and methods:**

A retrospective evaluation of postoperative CT scans and plain X-ray post-op images of 54 patients was analyzed using the OTOPLAN^®^ software. The post-op analysis was done for the angular insertion depth (AID) and center frequency of each electrode contact using both imaging modalities. Moreover, inter-rater reliability was assessed for measurements obtained from CT scans and plain X-ray images.

**Results:**

Non-significant statistical and clinical mismatches were detected when comparing the AID and center frequency measurements assessed using CT and X-rays. The absolute difference between CT and X-ray approaches ranged from 0.0 to 4.6 degrees for AID and 0.2 to 0.5 semitone for frequency. Moreover, the AID and the frequency measurements from CT and X-ray images demonstrated almost perfect agreement between the raters. The inter-observer reliability for CT scans showed that the intraclass correlation coefficient (ICC) exceeded 0.97 for AID and 0.95 for the frequency across all electrode contacts.

**Conclusion:**

Our results demonstrated the validity and reliability of using post-operative X-ray images by OTOPLAN^®^ software to build Anatomy-based Fitting maps.

## Introduction

Cochlear implantation (CI) stands as an effective treatment option for patients with severe to profound hearing loss that can significantly improve speech perception and communication [1]. The human cochleae exhibit a wide range of variations among individuals, mainly in the cochlear duct’s shape, length, and coiling pattern and even at the intra-cochlear level [2, 3]. These variations lead to the distinction of the tonotopic organization of sound frequency along the cochlea’s basilar membrane, which impacts the individuals’ sensitivity to specific frequencies and auditory perception [4]. Understanding the intricate anatomy of the cochlea plays an essential role in different aspects of the CI, such as surgical planning, electrode insertion and positioning, and post-operative programming.

The performance of CI recipients is markedly influenced by various factors such as the age at implantation, residual hearing and auditory function, device and surgical factors, and the quality of the CI processor programming [5]. One crucial aspect of the processor programming is the frequency allocation for each electrode contact. In current clinical practice, programming software typically relies on the default frequency fitting based on general population averages or standardized parameters [6]. However, this generalized default fitting approach does not account for the interindividual variation in cochlear anatomy, potentially resulting in a frequency-to-place mismatch [7].

Recent research has highlighted that incorporating anatomical variations in CI programming could potentially improve the outcome of the CI. It has been reported that implanted post-lingually deaf patients demonstrated better speech discrimination and rapid adaptation after employing anatomical mapping with subsequent frequency allocation [8].

OTOPLAN^®^ is a computer-based otological surgical planning software introduced by MED-EL (Innsbruck, Austria) in collaboration with CASCINATION AG (Bern, Switzerland). This software offers an easy way to analyze the anatomy of the temporal bone, estimating various cochlear metrics such as cochlear duct length (CDL) and angular insertion depth (AID) [9, 10]. This software plays a pivotal role in anatomy-based fitting (ABF) for CI, which involves tailoring frequency allocation to the individual’s unique cochlear anatomy, aiming to maximize the overall auditory performance [11]. To guide personalized programming using ABF, post-operative radiological imaging data should be imported to the OTOPALN^®^ and then to the programming software to assign a specific frequency range for each electrode contact based on its position inside the cochlea utilizing knowledge of the patient’s anatomy [12].

Computed tomography (CT) is considered a valuable tool for the preoperative assessment of CI and postoperative assessment of selected cases such as suspected malfunction of the device [13, 14]. However, routine post-operative CT scanning may be restricted by specific considerations, such as exposure to a hefty dose of ionizing radiation, which may be associated with potential risks for CI patients [15]. Additionally, it is costly, especially if not covered by health insurance. Furthermore, it is time-consuming, and children may move, which may cause artifacts. Fourth, there is difficulty in localizing the center point in each electrode. Fifth, it is done in the radiology department, which requires an appointment in most centers. These concerns about using post-operative CT put limitations on using ABF in routine clinical settings.

In contrast, post-insertion plain X-ray is routinely used to visualize the CI electrode position in the cochlea in many centers [16]. The first published results on ABF mapping using the post-insertion X-ray images are promising as they demonstrated the validity of this approach in AID measurements for frequency allocation in Comparison to CT-based measurements [17]. It is worth noting that in this study, the X-ray images were manually examined using the RadiAnt DICOM Viewer [17].

To date, no research has assessed the feasibility of analyzing the X-ray images with OTOPLAN^®^ software to obtain the AID and its corresponding center frequency. To address this gap, the current study aimed to assess the application of OTOPLAN^®^ software in performing post-op analysis on plain X-ray imaging by evaluating the AID and corresponding frequency for each electrode contact that would be used for ABF.

## Subjects and methods

This retrospective study commenced on 1 November 2023 after obtaining Institutional Review Board approval (IRB No. E-23-7997). Convenience sampling was used to select patients who fulfilled all the inclusion criteria, and data was retrieved from CI patients’ medical records that were presented to our tertiary cochlear implant center. Patient data was extracted anonymously; therefore, authors had no access to information that could identify individual participants.

### Patients’ selection

Based on reviewing the CI patients’ records, 50 patients from our Center, were enrolled in the present study. Our inclusion criteria were as follows: ears with normal cochlea that underwent cochlear implant surgery, with complete CI insertion, and those who performed postoperative temporal bone CT scans due to exceptional circumstances such as mastoiditis, swelling over the magnet, or exposure to trauma. The study included all patients who underwent post-operative plain X-ray imaging as a part of their clinical routine. Patients with cochlear malformation, no post-operative CT, insufficient CT imaging resolution, inner ear anomalies, and partial electrode insertions were excluded.

### Image analysis

Using both post-op CT scans and plain X-ray images (cochlear view), the manual approach of OTOPLAN^®^ 3.1.0 (V5) was used to calculate the AID and estimate the corresponding frequency for each electrode contact. These measurements were performed by two independent observers with the same level of training on the software, used the software professionally for at least three years, and have a solid understanding of the ear anatomy.

For CT images, the analysis was conducted as follows:

1. The post-op CT image was uploaded into OTOPLAN^®^ data management.
2. The observers manually defined the cochlear view, cochlear diameter (A value), width (B value), and height (H value).
3. Then, the software automatically calculated the cochlear duct length (CDL), the AID, and the corresponding center frequency for each contact.

As a next step, for plain X-ray images, the analysis was conducted as follows:

1. The post-op X-ray image was uploaded into OTOPLAN^®^ data management.
2. The observers manually defined the cochlear coordinates–round window and cochlear center points.
3. Once the electrode contact points were set manually, the software automatically calculated each contact’s AID and corresponding center frequency.

The mean of both raters’ measurements for each sample was used for further analysis.

### Sample size calculation

Assuming that the detected effect size between Ct and X-ray measured angular insertion depth (Cohen’s d for paired t-test, 1982) was 0.58, estimated from Alahmadi et al. study [17]. The required optimal sample size for paired CI patients at a 5% level of significance to achieve 90% power of the study will be 34 cases, adding to a 20% dropout rate (7 more cases) to get a total of 41 patients’ records required to conduct the study.

### Statistical analysis

Data collected for cochlear parameters, AID, and frequencies for each electrode contact were analyzed using R software version 4.2.2, "Innocent and Trusting." Descriptive statistics such as means, standard deviations (SD), and ranges were used for quantitative variables, whereas frequency and percentage were applied for categorical variables.

Intra-class correlation coefficient (ICC) was used to assess the inter-observer reliability between two independent readers for AID and frequency measurements obtained using CT scan and X-ray imaging. ICC values were interpreted based on the following: poor (<0.40), fair (0.40–0.59), good (0.60–0.74) and excellent (0.75–1.00). Furthermore, comparative analyses between measured AID and frequency using CT scan and X-ray were carried out using paired t-test, or Wilcoxon signed rank test, according to the normality assumption tested using the Shapiro–Wilk test. Significance was determined by P values less than 0.05 and a Confidence Interval (CI) of 95%.

## Results

The study was conducted on 50 cochlear implanted ears; 56.0% were right-sided, and 44.0% were left-sided. The mean age was 16.9 ± 12.5 years, and the mean age at implantation was 9.0 ± 12.6 years old, with males representing about 60.0% and females 40.0%. Notably, 72.0% of cases were implanted with FLEX28, and about 28.0% used the FORM24 electrode array (MED-EL, Innsbruck, Austria). The average cochlear parameters measured values were 9.4 ± 0.6 mm for A-value, 7.0 ± 0.5 mm for B-value, 3.7 ± 0.3 mm for H-value, and 36.9 ± 2.4 mm for CDL as detailed in Table 1.

**Table 1:** Baseline patients’ characteristics.

Demographics		Overall (N = 50)
**Gender**	Female	20 (40.0)
	Male	30 (60.0)
**Type of electrode**	Flex 28	36 (72.0)
	Form 24	14 (28.0)
**Age (years)**	Mean (SD)	16.9 (12.5)
	Min—Max	6.1–55.0
**Age at implantation (years)**	Mean (SD)	9.0 (12.6)
	Min—Max	0.7–49.2
**Side**	Left	22 (44.0)
	Right	28 (56.0)
**A value (mm)**	Mean (SD)	9.4 (0.6)
	Min—Max	7.90–11.09
**B value (mm)**	Mean (SD)	7.0 (0.5)
	Min—Max	5.93–8.22
**H value (mm)**	Mean (SD)	3.7 (0.3)
	Min—Max	2.84–4.78
**CDL (mm)**	Mean (SD)	36.9 (2.4)
	Min—Max	32.24–43.87

### Inter-observer reliability for AID and frequency measurements obtained from CT scan and X-ray

The AID and the frequency measurements, assessed via CT by two independent readers, demonstrated almost perfect agreement. The inter-observer reliability while using CT scans evaluated by the ICC exceeded 0.96 for AID and 0.94 for the frequency across all twelve electrode contacts—similarly, the AID and frequency measured using X-ray images displayed almost perfect agreement between the two raters. While using X-ray images, the inter-observer reliability demonstrated ICC of at least 0.92 for the AID and 0.9 for the frequency across all electrode contacts (Table 2).

**Table 2:** Intra-class correlation coefficients for angular insertion depth (AID) and frequency measured by X-ray and CT across all electrode contacts.

Measurements	Contacts	ICC between 2 CT readers	ICC between 2 X-ray readers
**Angular insertion depth (Degree)**	1^st^ electrode	0.984 (95%CI: 0.972 to 0.991)	0.987 (95%CI: 0.978 to 0.993)
	2^nd^ electrode	0.983 (95%CI: 0.970 to 0.991)	0.982 (95%CI: 0.968 to 0.989)
	3^rd^ electrode	0.981 (95%CI: 0.965 to 0.989)	0.985 (95%CI: 0.972 to 0.992)
	4^th^ electrode	0.980 (95%CI: 0.966 to 0.989)	0.969 (95%CI: 0.947 to 0.982)
	5^th^ electrode	0.992 (95%CI: 0.987 to 0.996)	0.957 (95%CI: 0.926 to 0.976)
	6^th^ electrode	0.987 (95%CI: 0.977 to 0.992)	0.958 (95%CI: 0.927 to 0.976)
	7^th^ electrode	0.980 (95%CI: 0.965 to 0.988)	0.947 (95%CI: 0.908 to 0.970)
	8^th^ electrode	0.970 (95%CI: 0.948 to 0.983)	0.944 (95%CI: 0.904 to 0.968)
	9^th^ electrode	0.967 (95%CI: 0.942 to 0.981)	0.942 (95%CI: 0.898 to 0.967)
	10^th^ electrode	0.972 (95%CI: 0.952 to 0.984)	0.946 (95%CI: 0.903 to 0.970)
	11^th^ electrode	0.981 (95%CI: 0.967 to 0.989)	0.928 (95%CI: 0.876 to 0.959)
	12^th^ electrode	0.967 (95%CI: 0.943 to 0.981)	0.917 (95%CI: 0.853 to 0.953)
**Frequency (Hz)**	1^st^ electrode	0.986 (95%CI: 0.976 to 0.992)	0.991 (95% CI: 0.984 to 0.995)
	2^nd^ electrode	0.973 (95%CI: 0.952 to 0.985)	0.982 (95% CI: 0.969 to 0.990)
	3^rd^ electrode	0.949 (95%CI: 0.911 to 0.971)	0.985 (95% CI: 0.971 to 0.992)
	4^th^ electrode	0.957 (95%CI: 0.926 to 0.976)	0.970 (95% CI: 0.947 to 0.983)
	5^th^ electrode	0.985 (95%CI: 0.974 to 0.992)	0.958 (95% CI: 0.928 to 0.976)
	6^th^ electrode	0.987 (95%CI: 0.977 to 0.993)	0.959 (95% CI: 0.928 to 0.976)
	7^th^ electrode	0.970 (95%CI: 0.948 to 0.983)	0.947 (95% CI: 0.907 to 0.969)
	8^th^ electrode	0.962 (95%CI: 0.934 to 0.978)	0.934 (95% CI: 0.887 to 0.962)
	9^th^ electrode	0.958 (95%CI: 0.928 to 0.976)	0.932 (95% CI: 0.881 to 0.961)
	10^th^ electrode	0.957 (95%CI: 0.925 to 0.975)	0.940 (95% CI: 0.891 to 0.966)
	11^th^ electrode	0.970 (95%CI: 0.947 to 0.983)	0.919 (95% CI: 0.861 to 0.953)
	12^th^ electrode	0.942 (95%CI: 0.90 to 0.966)	0.90 (95% CI: 0.822 to 0.943)

### Comparative analysis of AID and frequency measurements obtained from CT scan and X-ray

**Fig 1:**
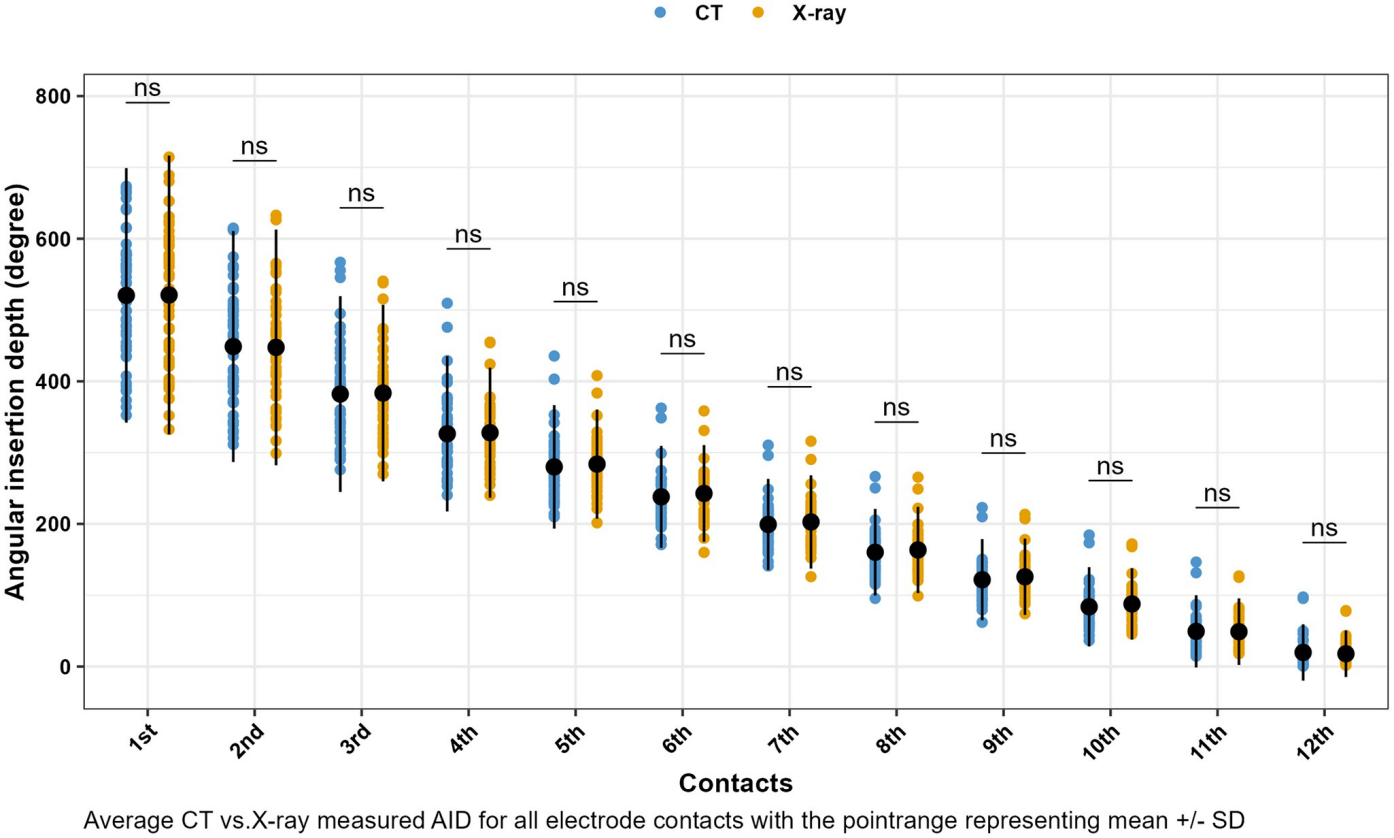
CT vs. X-ray measured angular insertion depth (AID) for all electrode contacts. Represented as means, and error bars represent the standard deviations.

**Fig 2:**
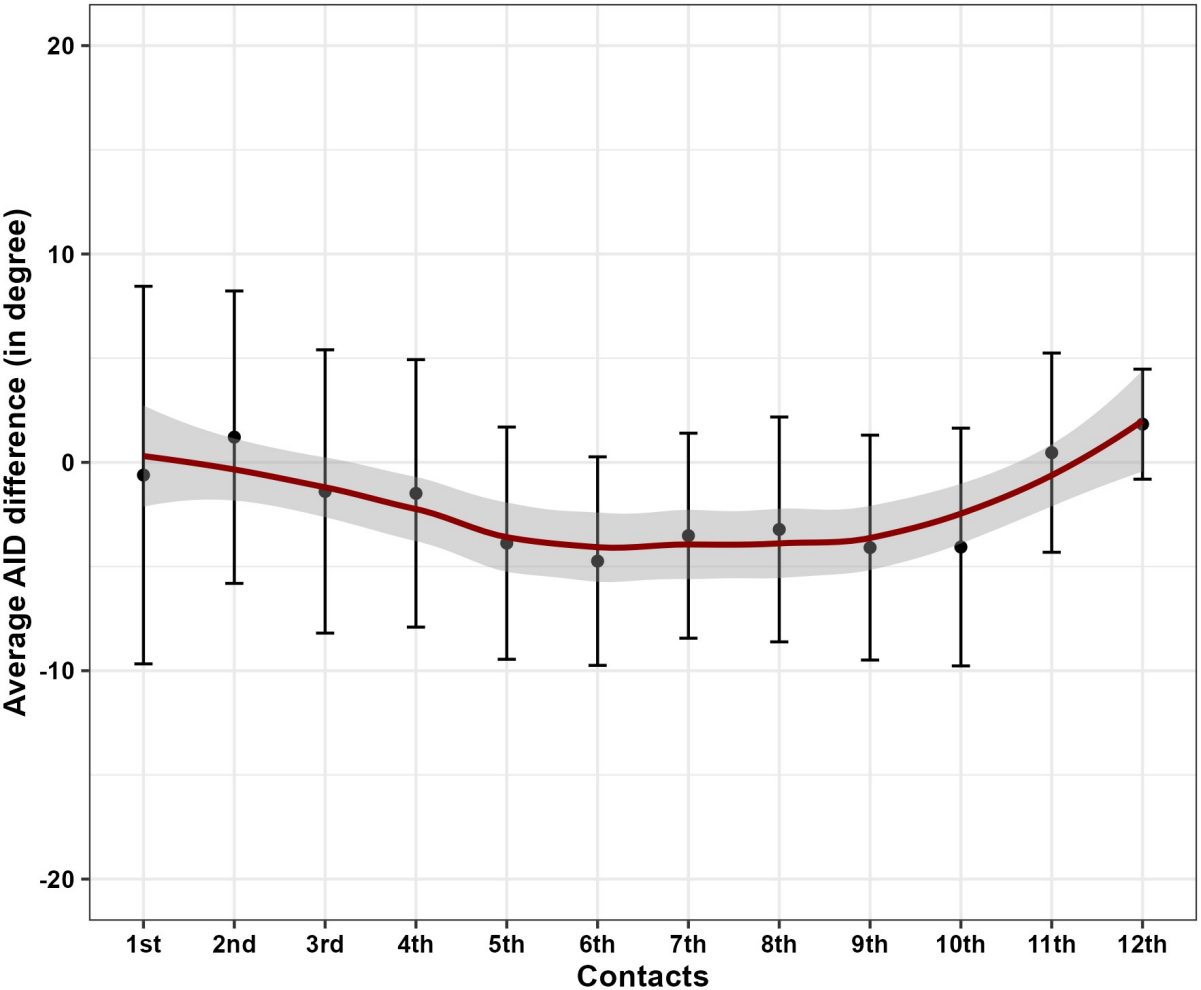
Average differences between CT and X-ray measured angular insertion depth (AID) for all electrode contacts. Represented as means and error bars represented the 95% Confidence Intervals.

The mean AIDs measured by CT and X-ray across all electrode contacts are summarized in Table 3 and depicted in Figs 1 and 2. The absolute difference between both approaches ranged from 0.47 to 4.74 degrees, indicating non-significant mismatching (p values > 0.05) across all electrode contacts.

**Table 3:** Comparative analysis between CT and X-ray measured angular insertion depth (AID) and frequency for all electrode contacts.

Methods	1^st^ electrode	2^nd^ electrode	3^rd^ electrode	4^th^ electrode	5^th^ electrode	6^th^ electrode	7^th^ electrode	8^th^ electrode	9^th^ electrode	10^th^ electrode	11^th^ electrode	12^th^ electrode
**AID (degree)**	520.5 (89.3)	448.8 (81.0)	382.2 (68.6)	326.5 (54.4)	280.0 (43.3)	238.0 (35.7)	199.3 (31.9)	160.3 (30.4)	121.8 (28.5)	83.8 (27.8)	49.3 (25.3)	19.7 (19.8)
**CT, *mean (SD)***												
**X-ray, *mean (SD)***	521.1 (97.7)	447.6 (82.7)	383.6 (61.9)	327.9 (45.6)	283.9 (38.3)	242.7 (33.9)	202.8 (32.7)	163.5 (30.2)	125.9 (26.8)	87.8 (25.0)	48.9 (23.4)	17.9 (16.3)
**Difference (95% CI)**	-0.61 (-9.67, 8.45)	1.21 (-5.81, 8.23)	-1.40 (-8.19, 5.40)	-1.49 (-7.91, 4.93)	-3.88 (-9.45, 1.69)	-4.74 (-9.74, 0.27)	-3.52 (-8.44, 1.40)	-3.22 (-8.61, 2.18)	-4.09 (-9.49, 1.31)	-4.06 (-9.77, 1.64)	0.47 (-4.31, 5.25)	1.83 (-0.81, 4.47)
**P value**	**0.893**	**0.731**	**0.824**	**0.466**	**0.198**	**0.094**	**0.241**	**0.521**	**0.291**	**0.162**	**0.996**	**0.358**
**Frequency (Hz)**	429.9 (226.1)	635.5 (278.1)	903.3 (320.0)	1229.5 (362.1)	1625.0 (403.3)	2126.6 (457.5)	2754.9 (553.1)	3627.0 (744.6)	4856.0 (1007.3)	6673.8 (1427.9)	9139.0 (1892.1)	12227.8 (2052.1)
**CT, *mean (SD)***												
**X-ray, *mean (SD)***	438.0 (253.5)	640.7 (281.2)	885.0 (300.3)	1199.6 (321.7)	1572.5 (375.4)	2056.5 (456.7)	2694.5 (580.5)	3541.4 (724.1)	4682.1 (899.0)	6396.7 (1245.2)	9139.0 (1784.2)	12380.6 (1815.9)
**Semitone mean (SD)**	0.19 (3.47)	-0.08 (2.44)	0.17 (2.36)	0.22 (2.36)	0.48 (2.22)	0.56 (2.06)	0.42 (2.07)	0.41 (2.40)	0.58 (2.60)	0.65 (3.05)	-0.05 (2.82)	-0.29 (1.68)
**P value**	**0.643**	**0.662**	**0.240**	**0.167**	**0.054**	**0.093**	**0.213**	**0.239**	**0.104**	**0.107**	**1.0**	**0.643**

**Fig 3:**
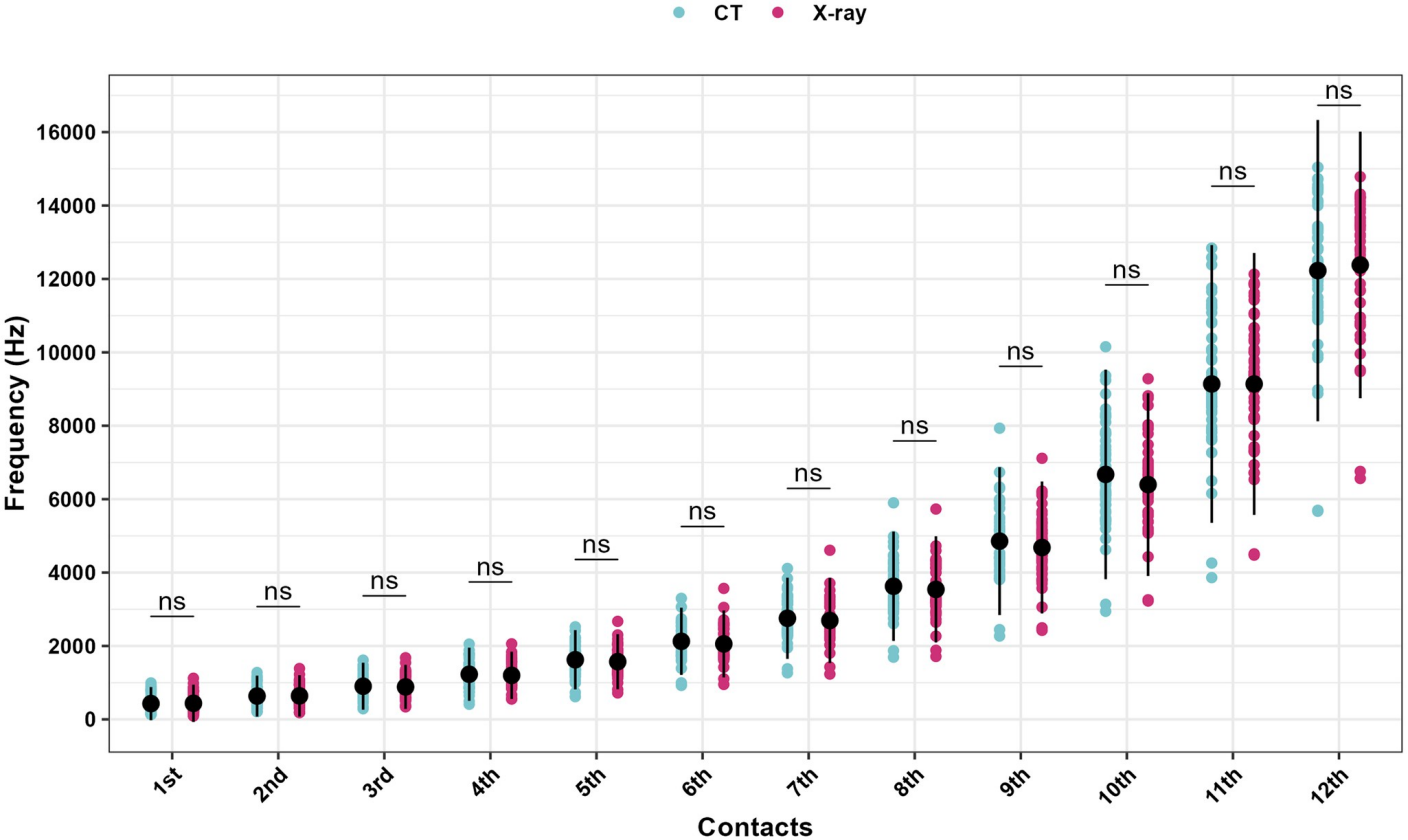
CT vs. X-ray measured frequency for all electrode contacts. Represented as means, and error bars represent the standard deviations.

**Fig 4:**
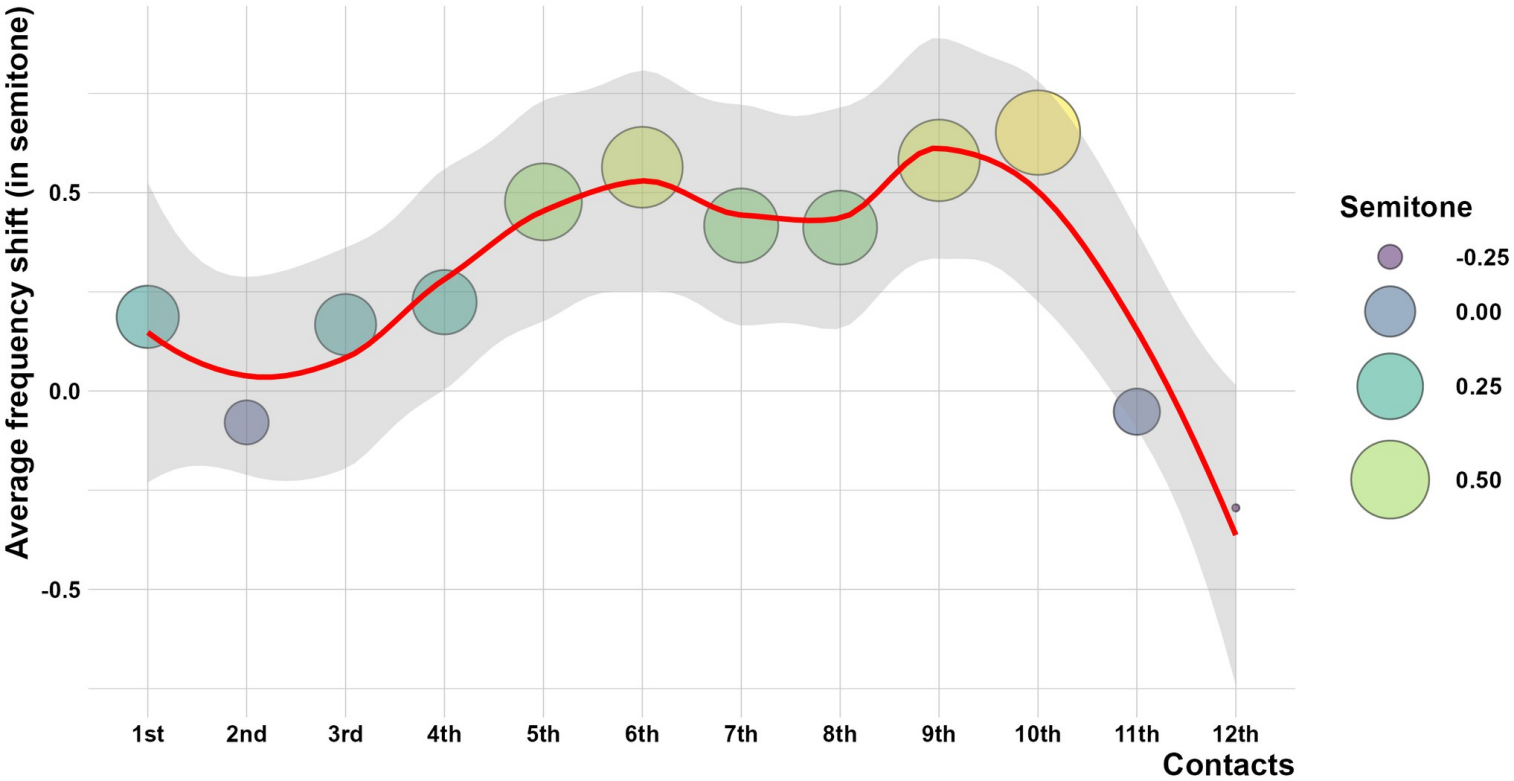
Bubble chart for average differences between CT vs. X-ray measured frequency (in semitones) for all contacts. Showing the best-fitted line with a 95% Confidence Interval.

When assessing the frequency across the twelve-electrode contacts, the average frequency errors between measurements obtained from CT and X-ray ranged from -0.29 to 0.65 semitones, indicating non-significant mismatching (p values > 0.05) across all electrode contacts as presented in Table 3, Figs 3 and 4.

## Discussion

OTOPLAN^®^ is a valuable tool for preoperative planning and post-operative electrode placement analysis of CI [18]. One of the principal uses of this planning software is to facilitate ABF, which aims to reduce frequency-to-place mismatch by ensuring precise electrode selection based on individual cochlear anatomy [19]. ABF holds promise for improving outcomes across a broad range of CI recipients, such as patients with cochlear malformation, residual hearing, or asymmetric hearing loss [18, 20]. Recent studies have reported better speech performance in patients with a more minor frequency-to-place mismatch [21, 22]. ABF for CI necessitates post-operative radiological evaluation, which is done using CT or X-ray imaging modalities. However, many considerations must be addressed to ensure that the radiological evaluation needed for ABF will benefit the CI recipients without compromising their health. The data presented herein support the validity and reliability of OTOPLAN^®^ software for first time the in analyzing post-operative X-ray images and in building ABF.

In the present study, OTOPLAN^®^ software was used for postoperative assessment of AID and frequency across the twelve-electrode contacts, using routine post-insertion X-ray images in Comparison to post-operative CT scans. Our results demonstrated that the inter-observer reliability of the AID based on post-operative CT and X-ray measurements was almost perfect, with an ICC exceeding 0.97 for CT and 0.92 for X-ray across all twelve electrode contacts. The reliability of this planning software in determining the AID of the array has been reported in an earlier study using post-operative CT scans [23]. Of note is that no previous study evaluated the feasibility of using this software in ABF using post-insertion X-ray images. The results of one previous study conducted by Alahmadi et al. were consistent with our results. However, their results depended on manually analyzing the post-insertion X-ray images using a DICOM viewer [17]. Our results demonstrated perfect agreement between the two readers in all twelve electrode contacts, which is closer to previously published findings [17].

In the present study, the absolute difference of AIDs measured by CT and X-ray ranged from 0.0 to 4.6 degrees, indicating non-significant mismatching across all electrode contacts. Interestingly, the C6 electrode contact exhibited the most remarkable absolute difference in AID measurements, with a value of 4.55 degrees between CT and X-ray approaches. These results agree with a previous study, which revealed that the absolute difference in AID measurements between the X-ray and CT was minimal for all electrodes (absolute mean difference of 4.7 degrees), with the highest difference at the C6 electrode [17]. This might be due to the slightly different appearance of electrode contacts when the electrode array approaches the first-to-second turn. However, further research might be suggested to gain valuable insights into possible reasons for the finding. In addition, Yoshimura et al. demonstrated a strong linear correlation between the AID measurements obtained from preoperative CT and those measured on post-operative X-ray [24]. Similarly, Fernandes et al. reported that X-rays and CTs analyzed manually provided an equivalent mean insertion depth with a mean difference of− 0.9 degrees [25]. Another study based on histological analysis of the cadaveric temporal bone model supported our findings, demonstrating that both radiographs and CTs can provide reasonably accurate angular depth of insertion measurements [26].

Next, OTOPLAN^®^ used the retrieved AID to calculate the corresponding frequency automatically. When assessing frequency, our results revealed that the frequency measurements assessed via CT and X-ray by two independent readers demonstrated almost perfect agreement with ICC = 0.95 for CT and 0.9 for X-ray across all twelve electrode contacts. In addition, we detected that the average frequency errors between measurements obtained from CT and X-ray ranged from -0.2 to 0.5 semitone, indicating neither statistically nor clinically significant mismatching across all electrode contacts. These findings align with the results of a previous study, which reported that the mean deviation in the absolute semitone between the mean of each electrode contact central frequency on CT and X-ray was (0.6 ± 0.4) with a median of 0.5 semitones [17].

Post-operative imaging of CI recipients is fundamental to providing information about the electrode position [27]. Although CT is highly accurate, it is associated with exposing patients to a considerable amount of ionizing radiation and has higher levels of metal artifacts [28]. It has been recommended that extensive postoperative radiological evaluation using CT for children should be avoided except for cases of malformation or any other complications [29]. On the other hand, using post-operative X-ray to determine electrode position is currently considered routine practice in clinics for uncomplicated cases, as it provides an accurate position inside the cochlea and reliable measurements for CI insertion depth [16, 27]. X-rays provide a lower-cost, lower-radiation option, which may be especially beneficial for pediatric patients more sensitive to ionizing radiation. However, X-rays have significant limitations when considering this approach in a clinical setting, including lower spatial resolution than CT scans, which may limit the capacity to detect some electrode-related problems. In addition, to ensure accurate measurements of the AIDs and corresponding frequencies, particular skull X-ray views have to be used, such as a modified Stenvers view (cochlear view) and Caldwell view [30]. Although these X-ray views are recognized as sufficient for evaluating the electrode insertion depth, the interpretations’ accuracy depends on the experience level of the radiology technicians and the readers [25, 31].

To our knowledge, this study is the first to assess the application of OTOPLAN^®^ software in ABF utilizing routine plain X-ray imaging. Our findings supported the idea that analyzing post-operative X-ray images with this software is a valid and reliable approach for ABF. However, it is essential to acknowledge that there are some limitations in the present study; one of them is that the discussed approach of ABF is only compatible with the electrode array of MED-EL. So, further research is needed to evaluate the applicability of ABF with other cochlear implant systems. In addition, only the manual option of OTOPLAN^®^ was used to analyze postoperative CT scans and X-ray images.
